# Global perspectives on practices and preferences in autologous free flap breast reconstruction: From flap selection to postoperative care A descriptive quantitative study

**DOI:** 10.1016/j.jpra.2024.10.010

**Published:** 2024-10-19

**Authors:** Sanderley J. Bonafastia, Lennart M. Steenbeek, Dietmar J.O. Ulrich, Stefan Hummelink

**Affiliations:** a Radboud University Medical Centre Nijmegen, Sanderley; b Radboud University Medical Centre Nijmegen, Lennart; c Radboud University Medical Centre Nijmegen, Dietmar; d Radboud University Medical Centre Nijmegen, Stefan

**Keywords:** Autologous breast reconstruction, Deep inferior epigastric perforator (DIEP) flap, Perioperative care, Perforator imaging, Flap monitoring

## Abstract

**Background:**

The purpose of this study was to evaluate the present-day practices in the preparation, peri-, and postoperative care for patients undergoing autologous free flap breast reconstructions (ABR) worldwide, with the aim of enhancing informed decision-making for plastic surgeons during the planning stages of ABR.

**Methods:**

A global survey was conducted among 280 plastic surgeons and 39 plastic and reconstructive surgery societies worldwide, enquiring about flap and donor site selection, surgical actions, perforator imaging, and perioperative care during ABR.

**Results:**

Eighty-two responses were received, among which 71% (n=58) were completed questionnaires. The preferred flap of choice was the deep inferior epigastric perforator flap (85%, n=51), with the internal mammary artery as the most commonly used recipient vessel. Preoperative imaging for ABR was typically performed using computed tomography angiography (75%, n=44) and often combined with a handheld Doppler. Handheld Doppler was the most frequently used modality to localize perforator vessels during surgery (33%, n=19), with the majority using either one (47%, n=24) or two (51%, n=26) perforators intraoperatively. These preferences were consistent across all clinic types.

Postoperatively, flap monitoring was primarily performed by the nursing staff, initially every hour on the first day and at reduced frequencies on subsequent days.

The most commonly used modality for monitoring flap viability was the handheld Doppler. The average length of hospital stay was 5 days.

**Conclusion:**

This study provides valuable insights into the current preparations and peri- and postoperative care in ABR procedures worldwide, aiding in the development of standardized practices and potentially improving patient outcomes.

## Introduction

Autologous “free flap” breast reconstruction (ABR) is a highly regarded option for women undergoing breast cancer surgery due to its natural feel, aesthetic appeal, and ability to maintain sensory function. The positive psychosocial benefits of this approach have been extensively acknowledged, making it a valuable option for a multitude of patients.^1^^,^^2^ In addition, ABR is a durable option that can endure the effects of aging, without the risk of implant-related complications or necessity for prothesis replacements.^3^ ABR procedures are now common in healthcare clinics worldwide, with various surgical methods described in the literature.^2^^,^^4^^,^^5^ Ongoing research into new imaging modalities and recovery optimization techniques aims to enhance the efficacy of these procedures.^6^^,^^7^ However, there is no centralized international consensus on ABR preparation and perioperative care, resulting in a dependence on nonstandardized national or intramural guidelines. Therefore, this study aimed to gain insight into the global practice patterns of ABR experts worldwide, focusing on flap choice, imaging modalities, monitoring devices, perioperative care, and additional surgical possibilities such as direct contralateral symmetrical reduction and surgical lymphedema treatment. The purpose of this study is to provide novice and experienced plastic surgeons with a global perspective on ABR practices, based on the collective insights from surgeons worldwide. This can serve as a reference point when implementing ABR in their hospitals or clinics, encouraging critical assessment, informed decision-making, and potential refinements of their local ABR guidelines.

## Method

This research is a descriptive quantitative study conducted by the Department of Plastic Surgery at Radboud University Nijmegen in the Netherlands. The aim was to gather information worldwide using a questionnaire consisting of 42 multiple-choice and 10 open-ended questions. The survey covered topics such as donor site selection, surgical approaches, imaging modalities, and perioperative care. Information about the participating surgeons’ details, practice settings, and experience were also collected. The survey questions are available in the appendix. The researchers extensively searched the PubMed and SciELO databases to identify plastic surgeons involved in articles related to ABR. A total of 280 subjects and 39 international societies of plastic and reconstructive surgery were contacted by mail for inclusion in this study. They were asked to complete an online survey using the LimeSurvey application (version 2.06+). Nonresponders were sent reminder emails 2 and 4 weeks after the initial email. If no response was received, a phone call was made to inquire about their interest in participating. This research targeted plastic surgeons who are directly involved with ABR procedures and those affiliated with healthcare centers where these procedures are performed.

The objective of this study was to collect data through an online questionnaire with subquestions directed to the respondents on the previous answers, assisting in providing an individual-centered questionnaire. Consequently, different sample sizes were taken for carrying out the analyses on specific segments of the data. The data were coded anonymously to ensure confidentiality and the analysis was carried out using IBM SPSS Statistics 27. Moreover, frequency analysis and crosstabulation tests were used to assess the demographics and relationship between multiple variables. Further, the Chi-squared test of independence was employed to determine the relationships between the categorical variables in the study. Although all responses were reviewed and analyzed, we prioritized presenting only the results with sufficient response rates. Data with low response rates or incomplete answers were excluded from the final analysis when they did not contribute meaningfully to the overall findings. This selective approach allowed us to focus on presenting reliable and representative trends, ensuring a more robust and comprehensive overview of global practices in autologous breast reconstruction.

## Results

A total of 82 responses were received, with 71% (n=58) being completed questionnaires. The tailored design of the study enabled respondents to respond to only questions that were applicable to them, resulting in varied sample sizes across subsections. Out of all 82 respondents who completed the demographic subsection, 59% (n=48) practiced only in an academic setting, 25% (n=21) in a nonacademic setting, 10% (n=8) in private clinics, 4% (n=3) in a both academic and private settings, and 2% (n=2) in all three settings.

Among these respondents, 33% (n=27) were from North America, 1% (n=1) from South America, 43% (n=35) from Europe, 19% (n=16) from Asia, and 4% (n=3) from Australia. Unfortunately, there were no respondents from Africa.

Among all the respondents, 74% (n=60) were microsurgeons performing ABR as principal surgeons and 75% (n=45) had less than 15 years of experience.

## Preoperative phase

### Preoperative imaging

Data from 60 respondents were analyzed to understand the prevalence and preferences of imaging techniques used in the preoperative phase. Among these respondents, approximately 75% (n=44) used preoperative imaging routinely, with most of them preferring CTA (82%, n=36). No association was observed between not using imaging and the unavailability of imaging modalities at the respondents’ healthcare centers. Among the 16 respondents who reported not using preoperative imaging, the majority (80%, n=12) stated that imaging would not make any difference in the outcomes.

Among the respondents who used imaging modalities preoperatively, 52% (n=23) used a combination of modalities. Sixty-one percent (n=14) used CTA with a handheld Doppler as their preferred combination, primarily to confirm the location of perforators and reduce intraoperative identification time. The use of a single imaging modality and a combination of modalities were evenly distributed across all healthcare centers: academic, nonacademic, and private clinics.

### Preventive actions

In the preoperative phase, insights were gathered from 77 respondents regarding preventive measures for perfusion problems. A substantial minority (41%, n=31) reported taking proactive preventive actions. Twenty-three respondents (74%, n=23) used low molecular weight heparin (LMWH), whereas 7 (23%, n=7) used mono-antiplatelet therapy.

## Intraoperative phase

### Donor site

Regarding intraoperative planning for ABR, data from 60 respondents were analyzed. Most respondents preferred to use abdominal donor site flaps, with the DIEP flap being the most commonly used (85%, n=51), followed by the transverse rectus abdominis myocutaneous (TRAM) flap (13%, n=8), and superficial inferior epigastric artery (SIEA) flap (2%, n=1). Among the 51 respondents who indicated having an alternative option, the SIEA- and the transverse upper gracilis flap were the most commonly used second-choice flaps, each used by 22% (n=13) of the respondents, followed by the TRAM flap in 17% (n=10) of the cases (Table 1).

**Table 1:** Respondents’ preferential donor site and their second choice donor site preferences in case their first choice was unavailable/unsuitable.

Donor site	Preferential donor site (%) (n=60)	Second choice (%) (n=51)
*Deep Inferior Epigastric Pedicle Flap (DIEP)*	85% (n=51)	7% (n=4)
*Transverse Rectus Myocutaneous Flap (TRAM)*	13% (n=8)	23% (n=14)
*Superficial Inferior Epigastric Artery Flap (SIEA)*	2% (n=1)	22% (n=13)
*Latissimus Dorsi Flap (LD)*	0%	2% (n=1)
*Thoracodorsal Artery Perforator Flap (TDAP)*	0%	2% (n=1)
*Superior Gluteal Artery Perforator Flap (SGAP)*	0%	5% (n=3)
*Inferior Gluteal Artery Perforator Flap (IGAP)*	0%	3% (n=2)
*Transverse Upper Gracilis Flap (TUG)*	0%	22% (n=13)
*Profunda Artery Perforator Flap (PAP)*	0%	7% (n=4)

### Recipient vessels

The internal mammary artery was the most commonly used recipient vessel (90%, n=54), followed by the thoracodorsal artery (82%, n=49) in case the internal mammary artery was unsuitable.

### Number of perforators

The distribution of perforators used was roughly even among the 51 respondents who preferentially used the DIEP flap: 47% (n=24) used one perforator and 51% (n=26) used two perforators. These preferences were consistent across academic, nonacademic, and private clinics.

### Intraoperative imaging

Data from 58 respondents were analyzed to examine the utilization trends of intraoperative imaging and their confidence levels in performing ABR. Approximately one-third (33%, n=19) used imaging to locate perforator vessels during surgery, with the majority (89%, n=17) using handheld Doppler. During ABR, 60% (n=35) of the respondents reported always feeling confident, while the remaining respondents indicated usually feeling confident during free flap harvesting. Notably, all respondents who reported feeling confident without intraoperative imaging usually used an imaging modality preoperatively.

The respondents also reported their confidence in flap harvesting without preoperative imaging. The confidence levels of the 43 respondents who completed the subsection on flap harvesting without preoperative imaging were analyzed and the majority reported moderate (28%, n=12) to very high (35%, n=15) levels of confidence. Respondents who reported always feeling confident in performing ABR used more frequent preoperative imaging (48%, n=28) than those who reported usually feeling confident (25%, n=15). However, no significant association was found between the confidence level during flap harvesting and preoperative imaging usage (p=0.21). The respondents’ confidence levels were self-estimated in this survey.

### Additional surgical possibilities

Data from 60 respondents were analyzed to identify the current intraoperative surgical methods in ABR surgeries. These procedures included contralateral symmetrical breast reduction, lymphedema treatment, and direct nipple reconstruction. Contralateral symmetrical breast reduction during initial ABR is not a standard procedure. One-third of the respondents (33%, n=20) never performed contralateral symmetrical breast reduction during the initial ABR, whereas approximately a quarter (23%, n=14) performed this in less than 25% of the cases. Approximately one-fifth (18%, n=11) always performed breast reduction in case of asymmetry. Furthermore, 8% (n=5) of the respondents performed it in more than 75% of the cases, 17% (n=8) in 25%-75% of the cases, and 3% (n=2) did so rarely or not at all. In academic and nonacademic settings, approximately half of the respondents performed contralateral reductions during the initial ABR, indicating a similar trend.

A minority of respondents (16%, n=10) treated lymphedema during ABR using vascularized lymph node transplantation (VLNT). All of them were working in an academic setting.

As for direct nipple reconstruction, the vast majority never performed this during the initial ABR (88%, n=53). There was no difference in the frequency of this supplementary procedure among academic, nonacademic, and private clinics.

### Prophylactic actions

The intraoperative preventive measures for perfusion issues were based on data from 77 respondents. Intraoperative preventive actions were reported by 43% (n=33) of the respondents. The most commonly reported actions included the application of warmth (42% n=14), followed by the administration of anticoagulant such as LMWHs (33%, n=11) and mono-antiplatelets (18%, n=6). Popular combinations included LMWH plus warmth application (18%, n=6) and LMWH plus mono-antiplatelet therapy (18%, n=6).

### Challenges and practice settings

**Figure 1:**
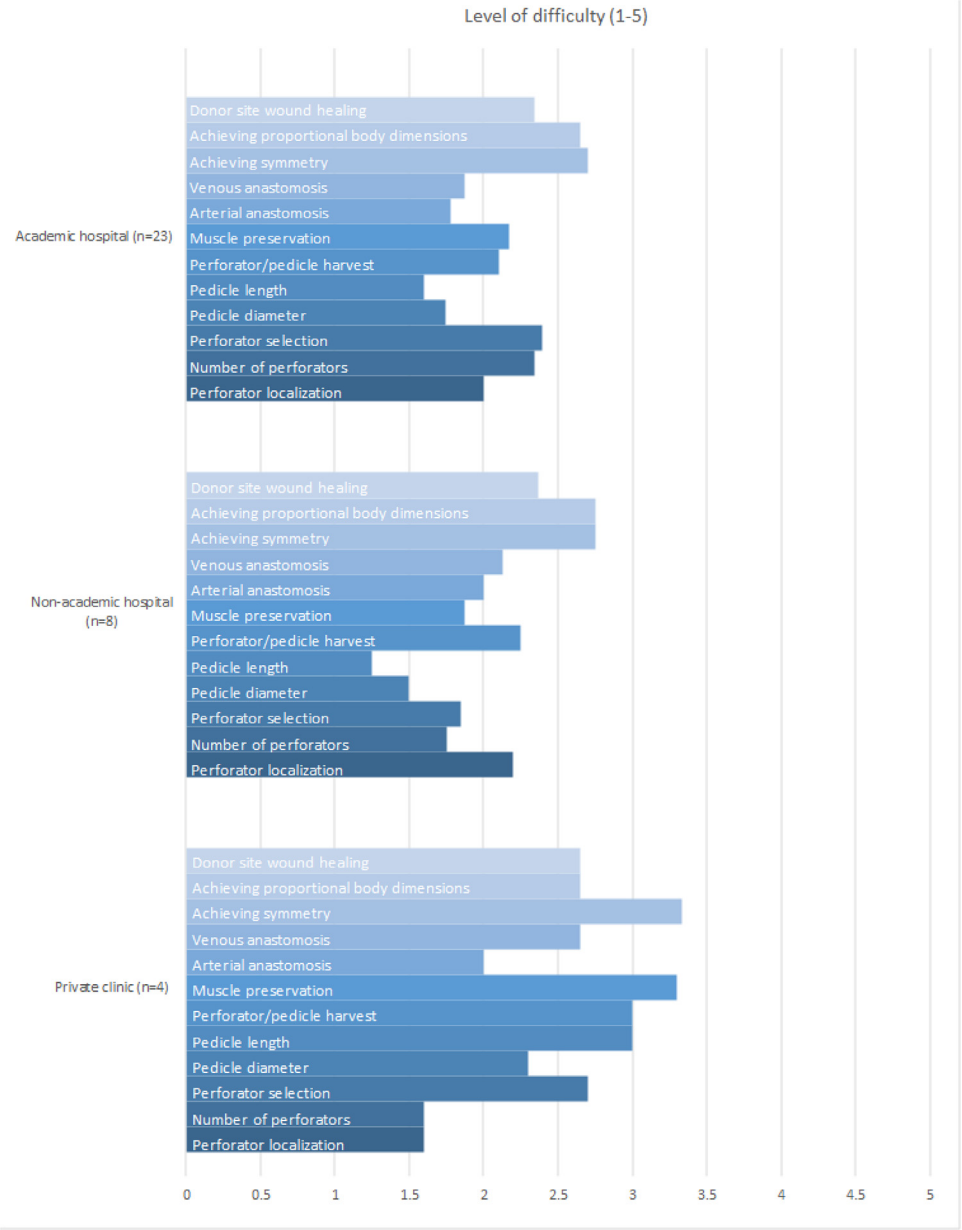
Level of reported difficulty in ABR in academic, nonacademic, and private clinics. The difficulty level is rated on a scale of 1 (not a challenge) to 5 (very challenging).

Intraoperative challenges were evaluated based on the data from the 58 respondents, highlighting the most challenging aspects of ABR. Respondents were asked to rate the challenges on a scale of 1 to 5, with 1 indicating “not a challenge” and 5 indicating “very challenging.” Among the 35 respondents who experienced challenges during ABR, achieving symmetry was rated as the most challenging, with a mean score of 2.8. Achieving proportional body dimensions in bilateral reconstructions was the second most challenging, with a mean score of 2.6, followed by donor site wound healing, which had a mean score of 2.4 (Fig. 1).

## Postoperative phase

### Flap failure

Flap failure and its management were analyzed based on the data from the 58 respondents, providing insights into the frequency and types of anastomotic revisions performed postoperatively. Among the 58 respondents, 79% (n=46) acknowledged experiencing postoperative flap failure to varying extents. In cases where anastomotic revisions were deemed necessary, 91% (n=42) of the respondents indicated the need to revise the venous anastomoses, while 76% (n=35) reported the need to revise the arterial anastomoses at various frequencies. The specific percentages and distribution of these revision frequencies are detailed in Table 2.

**Table 2:** Frequency with which venous or arterial anastomosis revisions were required when revision was deemed necessary.

Revision likelihood	Venous revisions	Arterial revisions
Rarely (1%-5% of cases)	28 (61%)	31 (68%)
Occasionally (6%-10% of cases)	7 (15%)	1 (2%)
Sometimes (11%-20% of cases)	0 (0%)	1 (2%)
Often (>20% of cases)	7 (15%)	2 (4%)
**Total respondents**	**42 (91%)**	**35 (76%)**

Notably, most respondents who experienced flap failure did not take preventive actions for perfusion problems preoperatively (60%, n=35) or intraoperatively (72%, n=42). However, no significant correlation was found between the incidence of flap failures and absence of preventive actions taken preoperatively (p=0.73) or intraoperatively (p=0.49).

## Postoperative phase

### Preventive actions

Postoperative preventive actions and their implications were assessed based on the data from 77 respondents. Among these respondents, 75% (n=58) reported taking postoperative preventive actions. Medicinal treatments were predominantly administered in the postoperative phase, with 67% (n=39) administering LMWHs and 28% (n=16) using mono-antiplatelet therapy. The most common combinations of preventive actions were LMWHs with warmth application (10%, n=6) or LMWHs with mono-antiplatelet therapy (10%, n=6).

### Flap viability assessment

Flap viability assessment was analyzed postoperatively based on the data from 72 respondents, offering insights into the frequency, methods, and personnel involved in the assessments. Flap viability was primarily monitored by ward personnel such as nurses alone in 86% (n=62) of the cases, together with a plastic surgeon in 19% (n=14), and alongside a plastic surgeon and resident in 15% (n=11) of the cases.

Monitoring was conducted hourly (67%, n=48) on the first day, every 2 h (49%, n=35) on the second day, and every 4 h (46%, n=33) on the third day after surgery. The viability of the flaps was assessed based on the following parameters: temperature (85%, n=61), color (97%, n=70), capillary refill (92%, n=66), and edema (53%, n=38). Most plastic surgeons (85%, n=61) used monitoring devices to assess flap perfusion. Handheld Doppler (74%, n=53) was the most commonly used device, followed by local SpO_2_ sensors (14%, n=10), implantable Dopplers (11%, n=8), and temperature sensors (11%, n=8).

### Hospital stays

**Figure 2:**
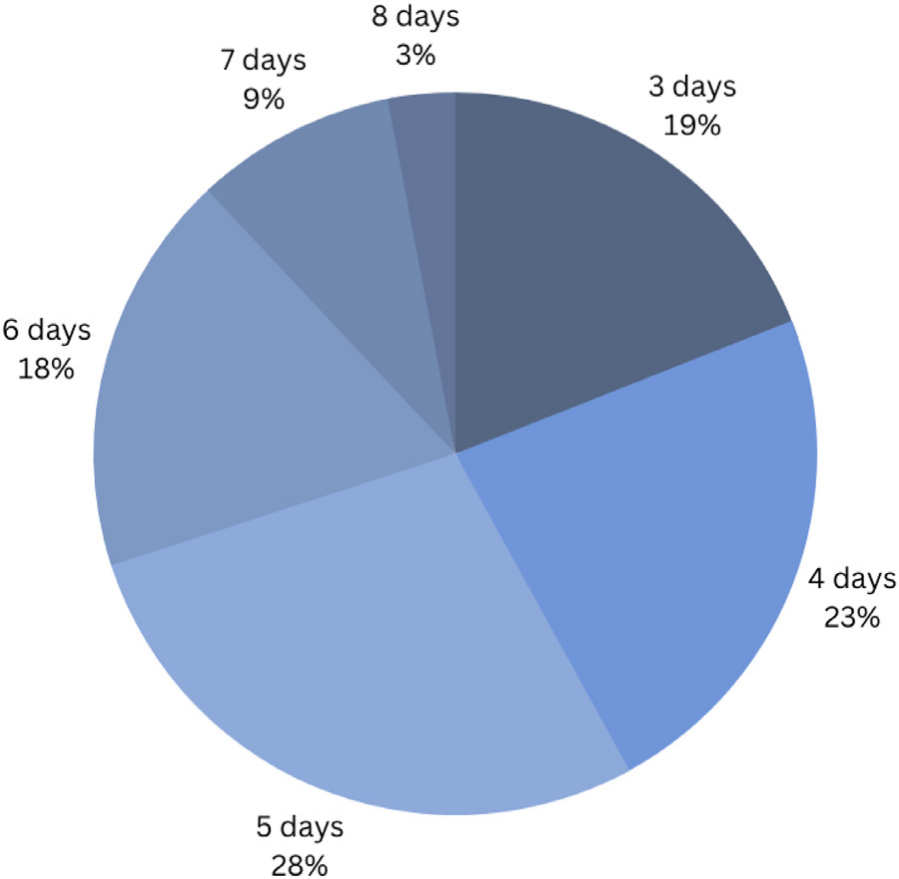
Duration of hospital stays for patients following autologous breast reconstruction in healthcare centers worldwide.

Data from 74 respondents were used to analyze the length of postoperative stay in the hospital. Sixty-eight percent (n=51) of the respondents discharge their patients within 5 days postoperatively (Fig. 2). The average length of hospital stay was 5 days for academic hospitals and private clinics and 4 days for nonacademic hospitals.

## Discussion

ABR is increasingly being considered as an option for women undergoing breast cancer surgery. This study assessed the current practices in ABR on a global scale. Despite the rising demand and technological advancements in ABR, the consensus on perioperative care remains elusive. Through a tailored survey approach, this research aimed to provide an overview of how ABR are performed worldwide.

A preoperative vascular map assists in selecting the appropriate perforator. Most plastic surgeons use CTA imaging as it has been shown to be the most accurate and precise method for visualizing perforators.^8^ Furthermore, recent studies have revealed that CTA imaging can also assess the suitability of flap tissue for mobilization.^9^ However, for patients who need to minimize their exposure to radiation or contrast, alternative imaging techniques such as MRA and handheld Doppler are valuable alternatives.^10^

In this study, most respondents (89%, n=17) used handheld Doppler alongside preoperative imaging to locate perforator vessels during surgery. The literature further identifies several real-time perforators imaging techniques, including duplex, fluorescence near-infrared angiography, and dynamic infrared thermography. Additionally, techniques such as image-guided stereotactic navigational systems and 3D-printed anatomical models are reported to enhance precision when paired with volumetric imaging.^6^

The reported usage rate of intraoperative imaging modalities was 33% (n=19), which was lower than initially anticipated.^10^ This discrepancy could be due to certain complementary techniques, such as the handheld Doppler, which were described and categorized as imaging modalities. Although the handheld Doppler is frequently used as a complementary technique to identify perforator vessels and assess anastomosis patency intraoperatively, it is technically not an imaging modality. Consequently, the reported use of “complementary techniques” might be understated compared to their actual usage.

Consistent with the findings of previous studies, this study's findings reaffirm that the abdomen remains the predominant donor site for microvascular ABR, with the DIEP flap often considered as the gold standard.^3^ A secondary benefit of selecting the abdominal donor site is the more aesthetic abdominal contour observed postoperatively.^11^ Notably, as with several studies, the choice of donor site remains a highly individualized decision, contingent on a patient's unique needs, circumstances, and body dimensions.

Regarding intraoperative challenges, muscle preservation during ABR was reported as a significant challenge, ranking it as the fourth most challenging aspect. Despite proper soft tissue management, this remains a critical issue.^7^ Although the literature claims limited donor site morbidity, clinically significant bulges or hernias are reported as complications of DIEP flap reconstructions.^12^ A promising robotic approach to DIEP flap harvest has been described in the literature, aiming to minimize abdominal wall disruption and optimize muscle preservation.^7^ Another major challenge in ABR is achieving proportional body dimensions and symmetry. Although the current techniques largely depend on the surgeon's judgment, a study by Hummelink et al. proposed a virtual flap planning method using 3D stereophotogrammetry and CTA, potentially assisting surgeons in accurately harvesting the correct flap volume.^5^

Regarding preventive actions against perfusion problems occurring postoperatively, 67% (n=39) of the surgeons administered LWMHs whereas 28% (n=16) used mono-antiplatelet therapy intraoperatively; a combination of both was observed in 10% (n=6) of the cases. LMWH has been described in the literature as an essential thromboprophylactic measure during surgery.^13^ Although LWMH is recommended, the use of a combination of LMWH and mono-antiplatelet therapy is debatable, especially for low-risk cardiovascular patients. Enajat et al., in their retrospective review, found no significant difference in the incidence of microvascular complications between patients who received both medications and those who received only LMWH perioperatively.^14^ Moreover, considering the known risks and significantly higher incidence of hematoma in patients receiving both medications, they recommended discontinuing the administration of mono-antiplatelet therapy postoperatively.

In cases of flap failure, venous anastomoses were revised more frequently (91%, n=42) than arterial anastomoses (76%, n=35), reflecting a higher likelihood of venous thrombosis over arterial occlusion as the cause of flap failure. Interestingly, a study conducted by Masoomi et al. revealed that venous thrombosis has a higher rate of successful treatment upon re-exploration compared to arterial occlusion.^15^ Given the urgency of timely interventions, 85% (n=61) of plastic surgeons use devices for postoperative neo-mamma monitoring, predominantly the handheld Doppler (74%, n=53), followed by local SpO_2_ sensors, implantable dopplers, and temperature sensors. With ongoing advancements, there is growing interest in innovative flap assessment methods. In recent literature, promising techniques such as near-infrared spectroscopy and implantable Doppler have been described for flap assessment, providing continuous objective physiological data on tissue perfusion.^16^^,^^17^

Regarding supplementary surgical interventions, a minority of respondents reported performing intraoperative lymphedema treatment using VLNTs. The limited implementation of this procedure may be due to it primarily being performed in academic settings and the logistical challenge of the additional operation time required. Nevertheless, the incidence of lymphedema following breast cancer treatment is relatively high, ranging from 24% to 49% after mastectomy.^18^ Research has shown that combining VLNT with DIEP flap breast reconstruction can significantly improve lymphedema-related quality of life rate.^19^ Regarding this supplementary procedure, this study focused solely on whether lymphedema treatment was performed during initial surgery. A further in-depth analysis of lymphedema treatment was not conducted.

Postoperatively, patients typically stay hospitalized for an average of 5 days. Prior research, including the one by Frey et al., associate microsurgical ABR with longer operative times and extended hospital stays.^20^ To address this, an enhanced recovery after surgery protocol has been introduced to optimize recovery of patients after DIEP flap reconstructions.^21^^,^^22^

In this study, 75% of the respondents had less than 15 years of experience as a surgeon. This suggests that the results represent the perspectives of relatively young and potentially recently trained group of surgeons. Furthermore, approximately three-quarters of the respondents were microsurgeons performing ABR procedures by themselves, indicating that the findings reflect current practices.

## Limitations

To ensure data accuracy for each subsection, all individual question responses were considered for each subsection, regardless of the survey completion status. Although this approach improved the understanding across topics, it led to varying sample sizes across the data subsections. To provide clarity, the specific sample sizes have been explicitly stated for each subsection. Additionally, the customized design of this study enabled respondents to answer only the questions that were applicable to them, contributing to the variability in sample sizes. Cross-continental comparisons were hindered by limited samples from different continents. This uneven distribution of data across continents highlights where ABR research or practices are the most common and also suggests potential geographical biases in the dataset. Accuracy in calculating the response rate was compromised by emails sent to individual plastic surgeons and societies of unknown sizes. Consequently, a reliable response percentage could not be determined and is therefore not reported.

Lastly, relying solely on self-assessments by respondents may introduce recall bias, especially regarding confidence in performing ABR or flap failure incidence. However, this approach also captures real-world perceptions and experiences, adding authenticity to the findings.

## Conclusion

This study provides valuable insights into the current practices of ABR worldwide, serving as a comprehensive overview for novice and experienced plastic surgeons. By broadening horizons beyond the local methodologies, it will aid in making well-informed decisions during the preparation and perioperative care of ABR, and underscores the potential areas for innovation in breast surgery. Although not a definitive guide, this study provides a state-of-the-art portrayal of how ABR is performed worldwide.

## Conflict of Interest

The authors declare no conflicts of interest as this study received no financial support, and the study report was not influenced by personal relationships of the authors.

## Appendix Supplementary materials

Image, application 1
