# Supplementary material for: Global perspectives on practices and preferences in autologous free flap breast reconstruction: From flap selection to postoperative care A descriptive quantitative study
Source: JPRAS Open. 2024 Oct 19;43:169–79. doi: 10.1016/j.jpra.2024.10.010 (PMC11697778; doi:10.1016/j.jpra.2024.10.010)
Supplement: Supplementary file 1 [file mmc1.docx]

**Appendix
Supplementary materials**

Questionnaire overview

1. Gender
   - [Male]
   - [Female]
2. In which setting do you routinely practice?
   - [Academic hospital]
   - [Non-academic hospital]
   - [Private Clinic]
   - [Other]
3. In which country do you practice?
4. In which city do you practice?
5. What is your primary specialty?
   - [General plastic surgery]
   - [Reconstructive surgery]
   - [General or trauma surgery]

- [Oncologic surgery]
- [Aesthetic surgery]
- [Trainee]

1. How many years of experience do you have as a (plastic) surgeon?

- [1-5 years]

- [6-10 years]

- [11-15 years]

- [16-30 years]

- [>30 years]

1. How many plastic surgeons practice in your department?
2. How many plastic surgeons perform autologous breast reconstructions in your clinic?
3. Do you perform free flap surgery for breast reconstruction yourself as principal surgeon?

- [Yes]

- [No]

1. For how many years have you been performing free flap breast reconstructions?
   - [<5 years]

- [5-10 years]

- [11-15 years]

- [16-25 years]

- [>20 years]

1. How much of your surgical time do you spend on free flap breast reconstructions?

- [10-25%]

- [25-50%]

- [51-75%]

- [>75%]

1. How many free flap breast reconstructions do you perform per year?
   - [<10]
   - [10-30]
   - [31-50]
   - [51-100]
2. Which free flap do you most frequently use for breast reconstruction?
   - [DIEP (Deep Inferior Epigastric Perforator)]
   - [SIEA (Superficial Inferior Epigastric Artery)]
   - [TRAM (Transverse Rectus Abdominus Myocutaneous)
   - [PAP (profunda artery perforator)]
   - [TUG (transverse upper gracilis)]
   - [ SGAP (superior gluteal artery perforator)]
   - [IGAP (inferior gluteal artery perforator)]
   - [LTP (Lateral Thigh Perforator)]
   - [ALT (Anterolateral Thigh)]
   - [Other]
3. How many of these do you perform annually?
   - [<10]

- [10-30]

- [31-50]

- [51-100]

1. How many perforators do you routinely include in these flaps?

- [1]

- [2]

- [3]

- [4]

- [5]

1. Which free flap is your second most frequently used procedure for autologous breast reconstruction?

- [DIEP (Deep Inferior Epigastric Perforator)]
- [SIEA (Superficial Inferior Epigastric Artery)]
- [TRAM (Transverse Rectus Abdominus Myocutaneous)
- [PAP (profunda artery perforator)]
- [TUG (transverse upper gracilis)]
- [ SGAP (superior gluteal artery perforator)]
- [IGAP (inferior gluteal artery perforator)]
- [LTP (Lateral Thigh Perforator)]
- [ALT (Anterolateral Thigh)]
- [Other]

1. How many of these second most frequently used free flap do you perform annually?
   - [<10]

- [10-30]

- [31-50]

- [51-100]

How many perforators do you routinely include in your second most frequently performed free flap for autologous breast reconstruction?
- [1]

- [2]

- [3]

- [4]

- [5]

1. Which vessel do you routinely use as a recipient site for free flap breast reconstruction?
   - [Internal mammary artery]
   - [Thoracodorsal artery]
   - [Serratus branch of subscapular artery]
   - [Lateral thoracic artery]
2. Which recipient vessel do you choose if your first choice isn't suitable?
   - [Internal mammary artery]
   - [Thoracodorsal artery]
   - [Serratus branch of subscapular artery]
   - [Lateral thoracic artery]
3. Is a free flap breast reconstruction reimbursed/covered by health insurances in your clinic?
   - [Yes, always]
   - [Yes, in most cases with some exemptions]
   - [Yes, but only in selected cases]
4. What are these exemptions?
5. Do you combine an unilateral free flap breast reconstruction with a contralateral symmetrical correction in one procedure?
   - [No, never]
   - [Yes, in < 25% of the cases]
   - [Yes, in 25-50% of the cases]
   - [Yes, in 51-75% of the cases]
   - [Yes, in >75% of the cases]
   - Yes, always if asymmetry occurs]
6. Do you perform the nipple reconstruction in the same operation as the free flap breast reconstruction?
   - [Yes, always]
   - [Yes, in >75% of the cases]
   - [Yes, in 51-75% of the cases]
   - [Yes, in the 25-50% of the cases]

- [Yes in <25% of the cases]
- [No, never]
- [Other]

1. Do you combine free flap breast reconstruction with surgical treatment of lymphedema of the arm?
   - [No]
   - [Yes, with vascularized lymph node transplantation (VLNT)]
   - [Yes, with microsurgical lymphovenous shunt]
   - [Yes, with lymphosuction]
2. Do you use any pre-operative imaging of the donor site routinely in the planning phase of free flap breast reconstructions?
   - [Yes]
   - [No]
3. What is the main reason not to do this?
   - [Selected cases only]

- [Specialist radiologist is not available]

- [Imaging modality of preference (Ct, MRI, etc.) is not available]

- [The information obtained is inadequate/unreliable]

- [It makes no difference to outcome]

- [No financial coverage]

1. Which modalities are available for you in your clinic?
   - [Magnetic Resonance Angiography (MRA)]
   - [Computed Tomography Angiography (CTA)]
   - [Handheld Doppler Ultrasound]
   - [Color Doppler Ultrasound/ Duplex]

- [Dynamic Infrared Thermography (DIRT)]
- [Indocyanine Green (ICG)]
- [Kent Imaging Camera]

1. Which modality do you routinely use pre-operative for donor site blood vessel localization/mapping?
   - [Magnetic Resonance Angiography (MRA)]
   - [Computed Tomography Angiography (CTA)]
   - [Handheld Doppler Ultrasound]
   - [Color Doppler Ultrasound/Duplex]
   - [Dynamic Infrared Thermography (DIRT)]
   - [Indocyanine Green (ICG)]
2. Do you combine this modality pre-operatively with an another technique?
   - [Yes, with handheld doppler ultrasound]
   - [Yes, with 3D printer]
   - [Yes, with augmented reality]
   - [Yes, with virtual reality]
   - [Yes, with a grid]
   - [Yes, with a projector]
3. What are the advantages of this combination (31)?
4. What are the disadvantages (31)?
5. Do you experience challenges in free flap breast reconstructive surgery in total?
   - [Yes]
   - [No]
6. What challenges do you experience?
   Please rank each items in a gliding scale from:  1 (=not a challenge) to 5 (=very challenging)
   - [Perforator localization]
   - [Number of perforators]
   - [Perforator selection]
   - [Pedicle diameter]
   - [Pedicle length]
   - [Perforator/pedicle harvest]
   - [Muscle preservation]
   - [Arterial anastomosis]
   - [Venous anastomosis]
   - [Achieving symmetry]
   - [Achieving proportional body dimensions (in bilateral reconstruction)]
   - [Donor site wound healing]
7. Are you confident in free flap harvesting?
   - [Always]
   - [Usually]
   - [Sometimes]
   - [Rarely]
8. How confident are you in harvesting the flap without pre-operative imaging?
   - [Totally]
   - [Very]
   - [Moderately]
   - [Slightly]
   - [Not at all]
9. Are you using modalities to localize perforator vessels intra-operatively?
   - [Yes]
   - [No]
10. Which modality do you use?
    - [Handheld Doppler Ultrasound]

- [Color Doppler Ultrasound/ Duplex]
- [Indocyanine Green (ICG)]
- [Dynamic Infrared Thermography (DIRT)]

- [Image-Guided Stereotactic Navigation]

- [Augmented Reality (e.g. glasses)]

- [Virtual Reality]

- [Handheld projector]

- [A grid]

1. Please describe how and when you use this modality during the operation.
2. How often is there a mismatch between pre-operative localization of the best perforator and your intra-operative findings?
   - [Always (>90%)]
   - [Usually (61-90%)]
   - [Sometimes (41-60%)]
   - [Rarely (10-40%)]
   - [Never (<10%)]
3. Do you monitor the flap post-operatively?
   - [Yes]
   - [No]
4. Who primarily monitors flap viability during the first few days post-operatively?

- [Plastic Surgeon]
- [Senior House Officer/ Specialist Registrar/ Resident]

- [Nurse]

1. How many times is the flap monitored after surgery?
   - [First day (24 hours) post-operative]

- [Second day post-operative]
- [Third day post-operative]

1. Which parameters are examined?
   - [Temperature]
   - [Color]
   - [Capillary refill time]

- [Turgor / Edema]
- [All of the above]

1. Which devices do you use for flap monitoring?
   - [None]
   - [Temperature sensor]
   - [Thermographic camera]
   - [Handheld Doppler Ultrasound]
   - [Implantable Doppler Ultrasound]
   - [Local SpO2 sensor (peripheral capillary oxygen saturation sensor)]
   - [Other]
2. Do you think this is the optimal way?
3. What improvements would you like to see?
4. Do you take actions pre-operatively to prevent free flap perfusion problems?
   - [No]
   - [Yes, mono antiplatelet therapy]
   - [Yes, dual antiplatelet therapy]
   - [Yes, low molecular weight heparins (LMWHs)]
   - [Yes, intravenous heparin by constant infusion pump]
   - [Yes, vitamin K antagonist]
   - [Other]
5. Do you take actions intra-operatively to prevent free flap perfusion problems?
   - [No]
   - [Yes, mono antiplatelet therapy]
   - [Yes, dual antiplatelet therapy]
   - [Yes, low molecular weight heparins (LMWHs)]
   - [Yes, intravenous heparin by constant infusion pump]
   - [Yes, vitamin K antagonist]

- [Yes, application of warmth in any way]
- [Other]

1. Do you take actions post-operatively to prevent free flap perfusion problems?
   - [No]

- [Yes, mono antiplatelet therapy]
- [Yes, dual antiplatelet therapy]
- [Yes, low molecular weight heparins (LMWHs)]
- [Yes, intravenous heparin by constant infusion pump]
- [Yes, vitamin K antagonist]
- [Yes, application of warmth in any way]
- [Other]

1. How often do you experience flap failure?
   - [Never]
   - [1-5%]
   - [6-10%]
   - [11-20%]
   - [>20%]
2. If perfusion problems occur, in how many cases do you have to revise your anastomosis?
   - [Venous anastomosis]

- [1-5%]
 - [6-10%]
 - [11-20%]
 - [>20%]

- [Arterial anastomosis]
 - [1-5%]
 - [6-10%]
 - [11-20%]
 - [>20%]

1. After how many days are most patients discharged from the hospital after a free flap breast reconstruction?
   - [After 3 days]
   - [After 4 days]
   - [After 5 days]
   - [After 6 days]
   - [After 8 days]
   - [After >8 days]
   - [Other]
